# Supplementary material for: Cognitive emotion regulation questionnaire: Evidence of internal structure through confirmatory factor modeling and exploratory structural equation modeling
Source: PLoS One. 2025 Jun 20;20(6):e0326319. doi: 10.1371/journal.pone.0326319 (PMC12180731; doi:10.1371/journal.pone.0326319)
Supplement: Attachment — Submitted filename: Response Letter.docx [file pone.0326319.s003.docx]

**Response Letter**

We would like to express our gratitude the editor for providing us with the opportunity to respond to the reviewer's comments. We also extend our sincere appreciation to both the editor and the reviewer for their time and effort in re-evaluating our manuscript. Below, we provide point-by-point responses to the comments, and the modifications made have been tracked in the manuscript.

**Reviewer #2:** Since the sample size has not been calculated, it is necessary to understand how the authors ensure that the sample is sufficiently large.

**Response:** Thank you. The suggested information has been added (see lines 86–92): “Although a prior calculation of the required sample size was not conducted, a sufficiently large sample was anticipated to ensure both the stability (i.e., that the estimation algorithm would converge on an admissible solution) and the accuracy of the parameter estimates (i.e., that the estimated parameter values would not substantially differ from the true population values) derived from the tested models (26,27). The simulation studies reported by Wolf et al. (26) suggest a minimum required sample size of 500 participants, considering the complexity of the models evaluated here (e.g., number of parameters to be estimated, number of items per factor).”

**Reviewer #2:** The eligibility criteria for the participants are still not presented.

**Response:** Thank you. This information can be found in lines 105-106: “It is important to note that, given the data collection method, there were no missing data, and the only inclusion criteria were agreeing to participate in the study and being 18 years of age or older.”

**Journal Requirements:**

Please review your reference list to ensure that it is complete and correct.

**Response:** Thank you. We have carefully reviewed the reference list to ensure it is complete and correctly formatted in accordance with the journal's requirements.

Thanks again, and we look forward to hearing from you.
